# Integrative Analysis Reveals Comprehensive Altered Metabolic Genes Linking with Tumor Epigenetics Modification in Pan-Cancer

**DOI:** 10.1155/2019/6706354

**Published:** 2019-11-07

**Authors:** Yahui Shi, Jinfen Wei, Zixi Chen, Yuchen Yuan, Xingsong Li, Yanyu Zhang, Yuhuan Meng, Yumin Hu, Hongli Du

**Affiliations:** ^1^School of Biology and Biological Engineering, South China University of Technology, Guangzhou 510006, China; ^2^Sun Yet-Sen University Cancer Center, State Key Laboratory of Oncology in South China, Collaborative Innovation Center for Cancer Medicine, Guangzhou 510060, Guangdong, China

## Abstract

**Background:**

Cancer cells undergo various rewiring of metabolism and dysfunction of epigenetic modification to support their biosynthetic needs. Although the major features of metabolic reprogramming have been elucidated, the global metabolic genes linking epigenetics were overlooked in pan-cancer.

**Objectives:**

Identifying the critical metabolic signatures with differential expressions which contributes to the epigenetic alternations across cancer types is an urgent issue for providing the potential targets for cancer therapy.

**Method:**

The differential gene expression and DNA methylation were analyzed by using the 5726 samples data from the Cancer Genome Atlas (TCGA).

**Results:**

Firstly, we analyzed the differential expression of metabolic genes and found that cancer underwent overall metabolism reprogramming, which exhibited a similar expression trend with the data from the Gene Expression Omnibus (GEO) database. Secondly, the regulatory network of histone acetylation and DNA methylation according to altered expression of metabolism genes was summarized in our results. Then, the survival analysis showed that high expression of *DNMT3B* had a poorer overall survival in 5 cancer types. Integrative altered methylation and expression revealed specific genes influenced by *DNMT3B* through DNA methylation across cancers. These genes do not overlap across various cancer types and are involved in different function annotations depending on the tissues, which indicated *DNMT3B* might influence DNA methylation in tissue specificity.

**Conclusions:**

Our research clarifies some key metabolic genes, *ACLY*, *SLC2A1*, *KAT2A*, and *DNMT3B*, which are most disordered and indirectly contribute to the dysfunction of histone acetylation and DNA methylation in cancer. We also found some potential genes in different cancer types influenced by *DNMT3B*. Our study highlights possible epigenetic disorders resulting from the deregulation of metabolic genes in pan-cancer and provides potential therapy in the clinical treatment of human cancer.

## 1. Introduction

Unlike normal cells that regulate their cell division reasonably, cancer cells have the ability to sustain growth-promoting signals, which lead them to obtain enough energy through adjusting epigenetics and metabolism mechanisms to meet the sustained cell division. In recent years, there has been a growing interest in cancer metabolism, especially the theory of carbohydrate metabolism disturbance which was proposed by Otto Warburg in the 1920s [1]. Besides glucose metabolism, other metabolism disorders including lipid and amino acid metabolisms have also been discovered in cancer [2–4]. In addition to providing energy, the research showed that disordered genes encoding metabolic enzymes may also promote tumorigenesis through other biological functions like epigenetic modification [5]. The most studied epigenetic alterations associated with carcinogenesis were variation in DNA and histone structure through posttranslational modifications and histone variants. Thereinto, DNA methylation and histone acetylation were the most common modifications related to the occurrence and development of cancer. Numerous studies have found that activation or overexpression of oncogene and silencing of cancer suppressor gene were due to the apparent epigenetic modification at their corresponding locations. The cancer suppressor gene *BRCA1* was found hypermethylated in ovarian cancer patients [6], and *CDH13* was methylated in endometrial carcinoma [7]. In the opposite case, hypomethylation may lead to the activation of normally silenced oncogenes. *RAS*, involved in cell differentiation, proliferation, apoptosis, cellular adhesion, and migration, was hypomethylated in the promoter in hepatocellular carcinoma [8].

Although separate investigation on these energy metabolism reprogramming and epigenetic modification dysfunction would uncover molecular characteristics, the diagnosis and cure effect that targeted them were not as expected [9, 10]. The metabolic network was observed to interplay with many altered signaling pathways in cancer, which increased the difficulty of developing targeted metabolic treatment [11]. In addition to oncogenic signaling, the extensive crosstalk was revealed between the metabolic networks and epigenetics disorders in cancer. For example, with the abnormal condition, acid environment or metabolic rewriting, epigenetic landscape would undergo dysfunction which contributed to gene expression disorder and neoplastic processes [12]. Metabolism has a significant influence on epigenetics through adjusting DNA and histone modification enzymes [13]; in turn, the epigenetics would affect the metabolism by altering the gene expression. These studies usually focused on the relationship between metabolism and epigenetics just in one type of disorder or in an isolated tumor type [14, 15]. In these specific analyses, the epigenetic alterations were a direct result of the altered activity of a particular metabolic enzyme. However, whether metabolic genes might contribute to epigenetic dysregulation more universally across multiple cancer types is not yet fully clear.

Given that anabolism and catabolism of metabolites and transcriptional dysregulation of metabolic genes are dramatically altered in cancer, there is an urgent issue to explore how such metabolic reprogramming alters epigenetic regulation at a global landscape. Researchers demonstrate that the gene expression patterns indeed reflected metabolic activities [16]. The projects such as the Cancer Genome Atlas (TCGA) provide large amounts of transcriptomic profiles across multiple tumor types with well-annotated human cancer samples [17], which is a good resource to explore metabolic dysfunction at the transcriptional level. The sufficient tumor and normal sample size with transcriptomic profiles are necessary for revealing relatively accurate metabolic dysfunction in each cancer. After searching the TCGA database by sample size for each cancer, we have performed a metabolic gene expression signature analysis covering 11 tumor types (normal samples of each cancer >30) including 5726 samples to identify critical metabolic gene features and their related epigenetic dysfunction, especially the DNA methylation. The different expression genes were also confirmed by Gene Expression Omnibus (GEO), NCBI's publicly available genomics database. To our knowledge, this is the first study that presents metabolic reprogramming plays a major role in the regulation of the epigenome across multiple cancer types. Our objective in this paper is to provide a global understanding of metabolic genes as well as their function in epigenetic modification in tumors and reveal potential targeted therapies for cancer.

## 2. Materials and Methods

### 2.1. Data Resources

Samples for different cancer types were downloaded from the TCGA data portal. To eliminate heterogeneity from patients and reveal universality more accurately, the minimum threshold of normal samples was 30 for each cancer type. After the screening, 5137 cancer and 589 normal samples were analyzed. 11 cancer types were selected as follows: breast invasive carcinoma (BRCA), colon adenocarcinoma (COAD), colorectal adenocarcinoma (COADREAD), head and neck squamous cell carcinoma (HNSC), kidney renal clear cell carcinoma (KIRC), kidney renal papillary cell carcinoma (KIRP), liver hepatocellular carcinoma (LIHC), lung adenocarcinoma (LUAD), lung squamous cell carcinoma (LUSC), stomach adenocarcinoma (STAD), and thyroid carcinoma (THCA). For each cancer type, we classified samples into 2 groups: normal samples and tumor samples (Supplementary Tables [Supplementary-material supplementary-material-1] and [Supplementary-material supplementary-material-1]).

For the validation material, we extracted the microarray expression profile (GEO group) from 2010 to 2019 in GEO database. Datasets were included if the following criteria were fulfilled: containing both tumor samples and adjacent normal tissues and normal sample size of more than 30. The five datasets (GSE13507, GSE87630, GSE37182, GSE76427, and GSE32665) containing adjacent normal tissues as the control were downloaded from the GEO repository. Details of each microarray study, including sample descriptions, are provided in Supplementary [Supplementary-material supplementary-material-1].

For this study, we used 2071 human metabolism-related genes assigned to metabolic pathways in the Cancer Cell Metabolism Gene DataBase (ccmGDB), official gene symbols, and gene IDs are included in Supplementary [Supplementary-material supplementary-material-1].

### 2.2. Differential Gene Expression Analysis

The mRNA expression profiles data were classified into 2 groups: normal and tumor. Gene expression was represented with TPM (transcripts per million) calculated by the counts of mapping reads in each gene. The gene with the mean TPM of less than 1 was excluded. The Cyber-T bayesreg.R, based on a Bayes-regularized unpaired *t* test, was used to analyze differences between tumor samples and normal samples. Differentially expressed genes (DEGs) were selected by FDR (false discovery rate) less than 0.05 and together with the absolute value of fold change value not less than 1.5. According to the metabolic gene list, we filtered the differential expression for each metabolic gene in each cancer type. For the GEO data, limma *R* package was used to screen the DEGs between tumor and normal samples in each included dataset. We performed gene differential analysis (|LogFC| > 1, adjusted *P* value (FDR) < 0.05) as the cutoff criteria. Hierarchical clustering analysis was performed for the DEGs using the *R* packages (“pheatmap”).

### 2.3. Functional Annotation

The metabolic differential expression genes (MDEGs) that were found to differentially express at least 8 cancers were selected. For functional annotation of those metabolic genes, we used GO and KEGG pathway annotations within David bioinformatics database to perform the gene ontology analysis [18, 19]. Significant pathways with a threshold of *P* < 0.05 were selected. The pathway enrichment bubble plot was drawn using *R* packages (“ggplot2”).

### 2.4. Survival Analysis

Kaplan–Meier analysis was used to identify the genes showing clinical relevance with tumor patients according to the TCGA datasets. The tumor samples were divided into two groups according to the median expression of each gene: high expression (with TPM values higher median) and low expression (with TPM values lower median). Then, the log-rank test was used to analyze the differences between groups, and the significantly prognostic value was selected with a threshold of the *P* value <0.05. Univariate Cox regression analysis was used to further identify the independent prognosis factors. Statistical analyses were performed using *R* package (“survival”).

### 2.5. DNA Methylation Analysis

For DNA methylation analysis, the average DNA methylation value (*β* values) for all CpG sites correlated with a gene in the genomic region between 0 and 1,500 bps ahead of the transcription start site was calculated. The ChAMP is an R/Bioconductor package that was used to calculate differentially the significantly expressed probes that had a *P* value <0.05 and |Δ*β*| > 0.2 between two group samples, respectively, in our analysis (http://www.bioconductor.org/packages/release/bioc/html/ChAMP.html). One group was between normal and tumor, and the other group was the different DNA methylated level between top10% and bottom10% samples by *DNMT3B* expression. Then, the overlap probes were obtained between the two groups. Differentially expressed probes with Δ*β* > 0.2 were defined as hypermethylation of the corresponding genes, and those with Δ*β* – 0.2 were defined as hypomethylation of the corresponding genes. Then, these probes were compared with different expression genes for the following analysis.

### 2.6. Spearman's Rank Correlation Analysis

Spearman's rank correlation was calculated using the function “cor.test” in *R* between promoter probe methylation level and their corresponding gene expressions. The significant negative correlations were considered if the correlation coefficient *r* was <0, and significant positive correlations were considered if *r* was >0. We calculated the above overlapped genes and their corresponding probes. The genes were obtained whose Δ*β* > 0.2 and *r* < –0.2 were regarded as hypermethylated and significantly anticorrelated between methylation and expression results.

### 2.7. Association Analysis between Oncogene/Tumor Suppressor Mutation and Metabolic Pattern

To identify the association between oncogene/tumor suppressor mutation events and metabolic profile, we first choose the top 15 mutation driver genes in pan-cancer according to the previous comprehensive study [20]. Information regarding mutation frequency and sample matrix in each cancer type was downloaded from the TCGA, an open-access database that is publicly available at cBioportal [21] (http://www.cbioportal.org). We choose the top 3 oncogene/tumor suppressor with a high mutation frequency for each cancer type and divided the sample into two parts according to whether each oncogene/tumor suppressor is mutated or not. The differential gene expression analysis was conducted between the above grouping samples in each cancer type. The associations were analyzed between the focus metabolic gene of this study as well the whole metabolic spectrum and key oncogene/tumor suppressor mutation.

## 3. Results

### 3.1. Global Changes in Metabolic Gene Expression

**Figure 1:**
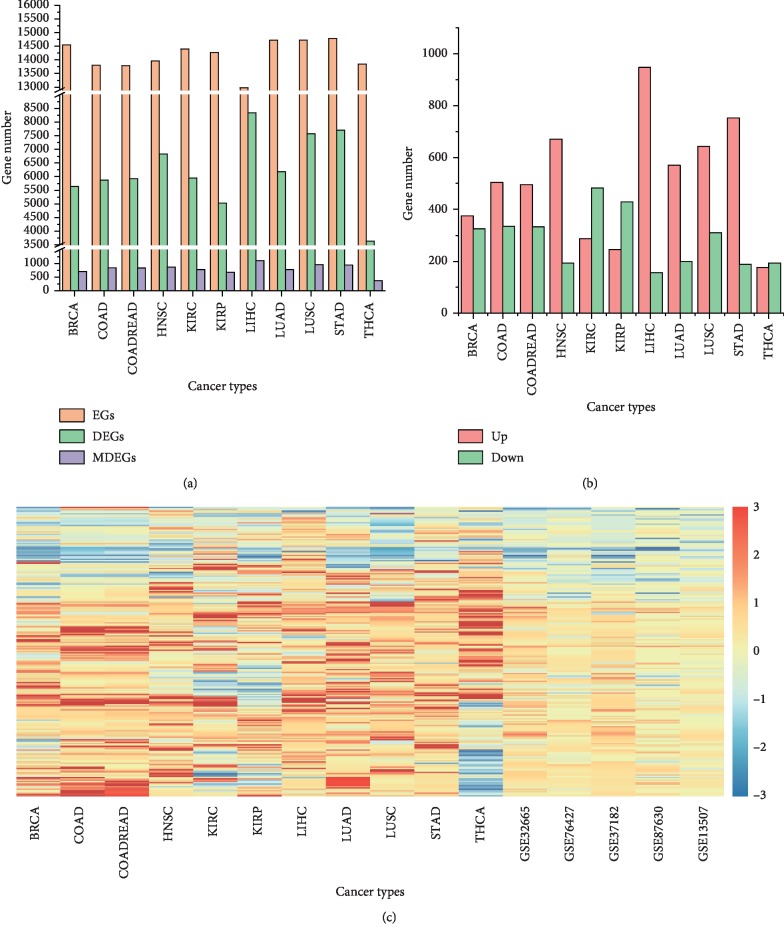
Cancer-induced changes in all gene and metabolic gene expressions. (a) The number of EGs, DEGs, and MDEGs in 11 different cancer types. Orange bar represents EGs, green bar represents DEGs, and purple bar represents MDEGs in 11 different cancer types. (b) The number of significantly upregulated and downregulation of MDEGs in 11 types of cancers. Significantly different genes were filtrated by the following criteria: FDR > 0.5, |Fold Change| > 1.5. Red and green bars represent upregulated and downregulation MDEGs, respectively, in 11 cancer types. (c) Heat map of differentially expressed genes in TCGA and GEO database. Red color represents the upregulated genes; blue color represents the downregulated genes; white color represents genes without change. EGs: expressed genes; DEGs: differentially expressed genes; MDEGs: metabolic differential expression genes; BRCA: breast invasive carcinoma; COAD: colon adenocarcinoma; COADREAD: colon rectum adenocarcinoma; HNSC: head and neck squamous cell carcinoma; KIRC: kidney renal clear cell carcinoma; KIRP: kidney renal papillary cell carcinoma; LIHC: liver hepatocellular carcinoma; LUAD: lung adenocarcinoma; LUSC: lung squamous cell carcinoma; STAD: stomach adenocarcinoma; THCA: thyroid carcinoma; TCGA: the cancer genome atlas; GEO: Gene Expression Omnibus.

**Figure 2:**
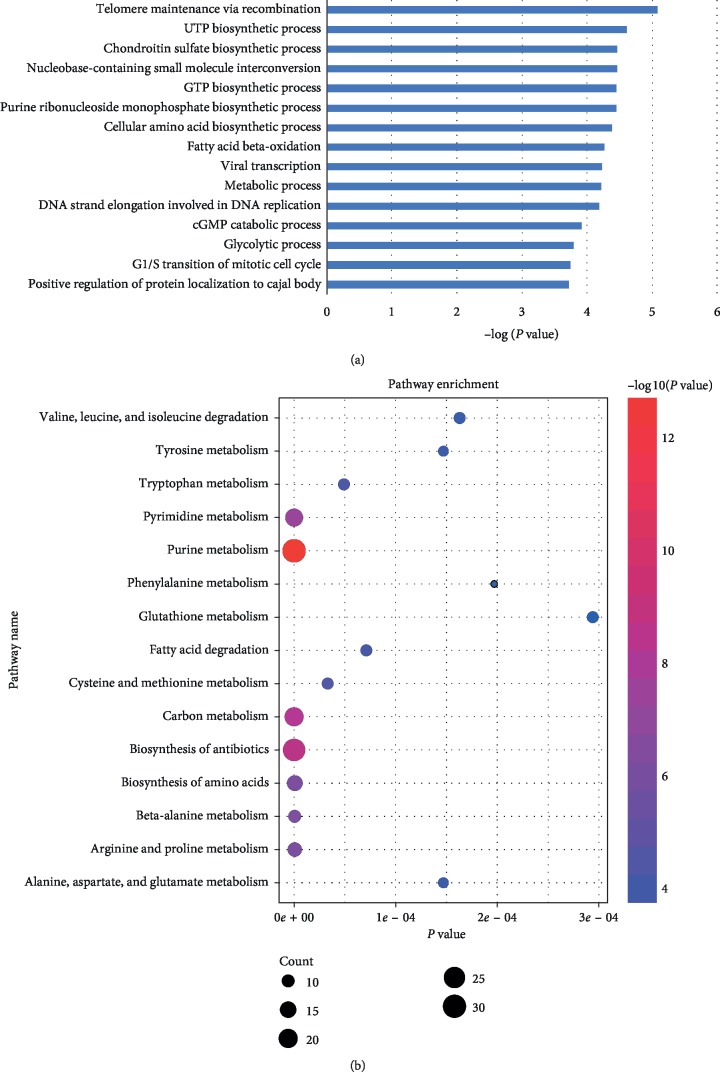
Gene ontology and pathway enrichment analysis of rewiring metabolic genes in cancer tissues compared to normal. (a) The gene ontology analysis; *P* < 0.01. (b) The KEGG pathway enrichment analysis; *P* < 0.01. The top 15 pathways were chosen and analyzed. The size of the circle indicates the number of enriched genes.

To understand metabolic gene expression in different cancers, we analyzed 5137 tumor and 589 normal samples spanning 11 different tumor types. According to the clinical information, we divided samples into 2 subgroups for each cancer type: normal and tumor (Supplementary [Supplementary-material supplementary-material-1]).  Figure 1(a) presents the total number of expressed genes (EGs), differentially expressed genes (DEGs), and metabolic differential expression genes (MDEGs) in 11 cancers. The number of upregulation and downregulation of MDEGs in 11 cancers is shown in Figure 1(b). To validate the different analysis results in TCGA, we choose the MDEGs (found differential expression at least 8 cancers) to get the differential distribution in GEO database. As shown in Figure 1(c), the GEO data exhibited the same differential variation trend as in TCGA cancer types. To gain a view of metabolic status in cancer, we carried out the MDEGs (were differentially expressed in at least 8 cancers) sets of metabolic pathways based on the GO and KEGG pathway analysis (Supplementary Tables [Supplementary-material supplementary-material-1] and [Supplementary-material supplementary-material-1]). The pathways, namely, purine metabolism, carbon metabolism, pyrimidine metabolism, and beta-alanine metabolism, were dysregulated in cancers (Figure 2, Supplementary [Supplementary-material supplementary-material-1]).

### 3.2. Disordered Metabolic Genes Related to Histone Acetylation

**Figure 3:**
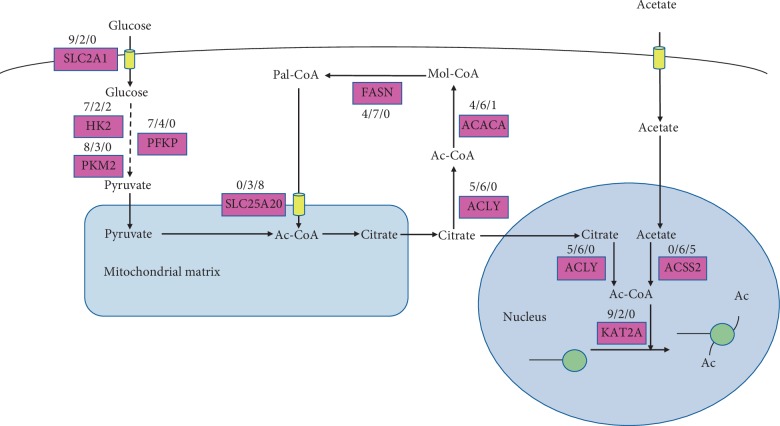
Detailed network map of transacetylation of gene expression levels in the 11 cancer types. Note: each metabolic reaction is marked with the number of tumors (out of 11 considered in our analysis) in which at least one gene in the corresponding reaction is significantly (FDR < 0.05) upregulated (red), downregulated (green), and neutral (black). If unmarked, no statistically significant change in mRNA expression was detected. Cytoplasmic citrate came from mitochondrial citrate which was produced by glucose for glycolysis in the cytoplasm and TCA cycling in the mitochondria. With the catalysis of ACLY, ACACA, and FASN, part of citrate was used to generate long-chain fatty acid which then would be oxidized in the mitochondria. Nuclear citrate and acetate were separately catalyzed by ACLY and ACSS2 to ac-CoA, which provided the acetyl groups to histones that led to histone acetylation. SLC2A1: solute carrier family 2 member 1; SLC25A20: solute carrier family 25 member 20; HK2: hexokinase 2; PKM2: pyruvate kinase M1/2; PFKP: phosphofructokinase, platelet; ACLY: ATP citrate lyase; ACACA: acetyl-CoA carboxylase alpha; FASN: fatty acid synthase; ACSS2: acyl-CoA synthetase short-chain family member 2; KAT2A: lysine acetyltransferase 2A; Ac-CoA: acetyl coenzyme A; Mol-CoA: malonyl-CoA; Pal-CoA: palmityl-CoA.

In the transacetylation process (Figure 3, Table 1), the citrate, acetate, and fatty acids were the main sources for generating acetyl coenzyme A (acetyl-CoA) which would provide the acetyl group to histone. In the citrate metabolism, the genes encoding pyruvate dehydrogenase (PDH) and ATP citrate lyase (ACLY) were included. *PDH* had no significant changes between cancer and normal samples. *ACLY* was upregulated in 5 cancer types. In the acetate metabolism, the gene-encoded acyl-CoA synthetase short-chain family member 2 (ACSS2) was downregulated in 5 cancer types. In the fatty acid synthetase part, *ACACA* and *FASN* were upregulated in 4 cancer types. In the fatty acid oxidation process, the acylcarnitine would be transported to the mitochondria to mediate fatty acid metabolism by solute carrier family 25 member 20 (SLC25A20). *SLC25A20* had a lower expression in 8 cancer types compared with those of normal samples. Citrate produced acetyl-CoA through the ACLY and acetate produced acetyl-CoA through ACSS2 also in the nucleus which indicated that acetyl-CoA existed in the nucleus and cytoplasm of mammalian cells, influencing both metabolism and the global regulation of the gene expression [5]. Histone acetyltransferases (HATs) could acetylate the acetyl group from acetyl-CoA to the histone. Lysine acetyltransferase 2A (KAT2A) was mainly studied as HATs. The gene *KAT2A* had an upregulated expression in 9 tumor types.

### 3.3. Disordered Metabolic Genes Related to DNA Methylation

**Figure 4:**
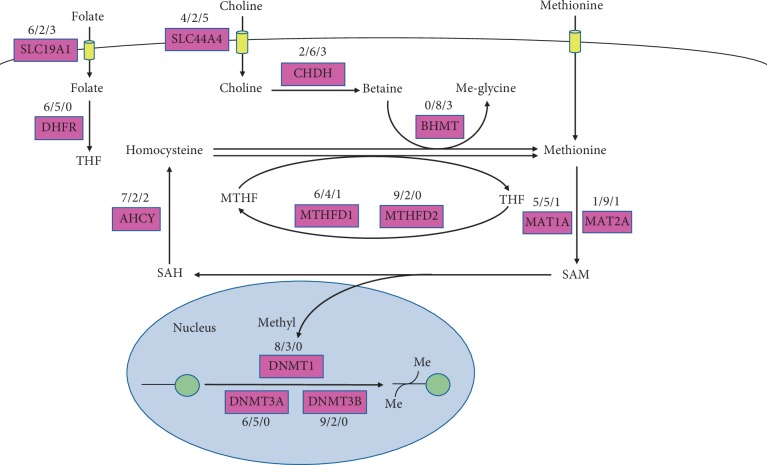
Detailed network map of transmethylation of gene expression levels in the 11 cancer types. Note: each metabolic reaction is marked with the number of tumors (out of 11 considered in our analysis) in which at least one gene in the corresponding reaction is significantly (FDR < 0.05) upregulated (red), downregulated (green), and neutral (black). If unmarked, no statistically significant change in mRNA expression was detected. There were three metabolites as methyl donors: methionine, folate, and betaine. All of them provided the methyl group to SAM through their metabolic cycle. Betaine was produced from choline by CHDH. The transporters of choline and folate were SLC44A4 and SLC19A1. Methionine and ATP were catalyzed into SAM by MATs. SAM donated its methyl group to DNA or histone in reactions catalyzed by MTs: DNMT1, DNMT3A, and DNMT3B. SAH was the byproduct of methylation reactions process from SAM. Then, SAH was regulated by the enzyme AHCY, which reversibly made this metabolite into adenosine and homocysteine. Homocysteine was used to produce methionine with the demethylation of folate and betaine through two enzymes: MS and BHMT. SLC19A1: solute carrier family 19 member 1; SLC44A4: solute carrier family 44 member 4; DHFR: dihydrofolate reductase; CHDH: choline dehydrogenase; BHMT: betaine-homocysteine S-methyltransferase; AHCY: adenosylhomocysteinase; MTHFD1: methylenetetrahydrofolate dehydrogenase, cyclohydrolase, and formyltetrahydrofolate synthetase 1; MTHFD2: methylenetetrahydrofolate dehydrogenase (NADP + dependent) 2, methenyltetrahydrofolate cyclohydrolase; MAT1A: methionine adenosyltransferase 1A; MAT2A: methionine adenosyltransferase 2A; DNMT1: DNA methyltransferase 1; DNMT3A: DNA methyltransferase 3 alpha; DNMT3B: DNA methyltransferase 3 beta; THF: tetrahydrofuran; MTHF: methyltetrahydrofolate; SAM: S-adenosyl-L-methionine; SAH: S-adenosyl-L-homocysteine.

The disordered metabolic genes in transmethylation pathways were filtered out in our results. Accordingly, we assembled a metabolic map depicting the distribution of these changes in the pathway (Figure 4, Table 1). There were three metabolites as methyl donors: methionine, folate, and betaine. All of them provided the methyl group to S-adenosylmethionine (SAM) through their metabolic cycle. In the betaine part, the expression of *SLC44A4*, *CHDH*, and *BHMT* was disordered across cancers and had a general downward trend. In the folate cycle, *SLC19A1* and *MTHFD1* had upregulated expression in 6, 6 tumors and downregulated expression in 3, 1 tumor, respectively. *DHFR* and *MTHFD2* were not downregulated in cancers, and they were upregulated in 6 and 9 cancers, respectively. Methionine and ATP were catalyzed into SAM by methionine adenosyl transferase (MAT). The gene *MATIA* was downregulated, and *MAT2A* was upregulated in one cancer. In the transmethylation reactions, the genes encoding histone methyltransferase (HMT) were not significantly different in cancers. Only DNA methyltransferase (DNMT) genes were presented in results: *DNMT1*, *DNMT3A*, and *DNMT3B* were all highly expressed in 8, 6, and 9 cancer samples compared with normal, respectively.

### 3.4. Survival Analysis of Disordered Metabolic Genes

**Figure 5:**
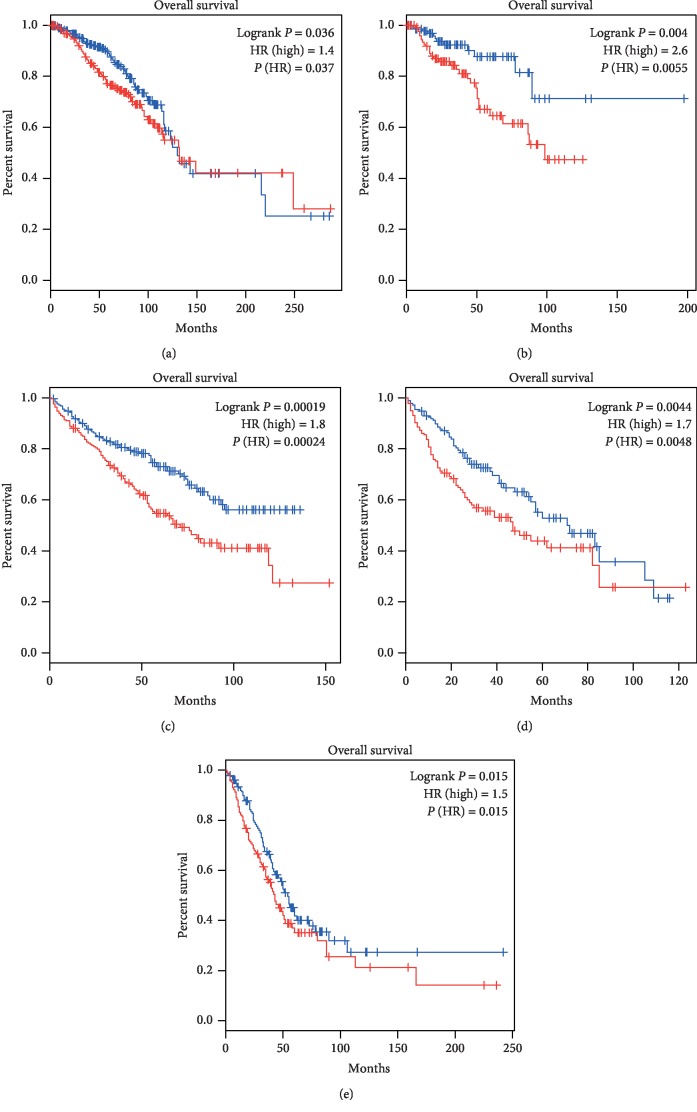
Survival analysis of gene *DNMT3B*. (a) BRCA, (b) KIRP, (c) KIRC, (d) LIHC, and (e) LUAD.

To analyze the DNA methylation and identify the related genes which may show clinical relevance with tumor patients, we performed the survival analysis on the disordered DNMTs genes using TCGA datasets. Disordered *DNMT3B* was screened and was found to have an impact on overall survival months. In BRCA, KIRC, KIRP, LIHC, and LUAD, patients whose tissues have a higher expression of *DNMT3B* had significantly shorter overall survival compared to those with lower expression. The higher expression of *DNMT3B* led to poor prognosis in those cancer types, especially in KIRP with the maximum hazard ratio (Figure 5). The *DNMT1* was significantly associated with patient survival times only in one cancer type (not shown), and it was seen that the *DNMT3A* expression did not affect patient survival.

The red plots present the high expression of each individual while the blue plots present the low expression of each individual. *P* value of less than 0.05 was considered as statistically significant. HR: hazard ratio.

### 3.5. *DNMT3B* Contributing to the Altered DNA Methylation and Corresponding Gene Expression

**Figure 6:**
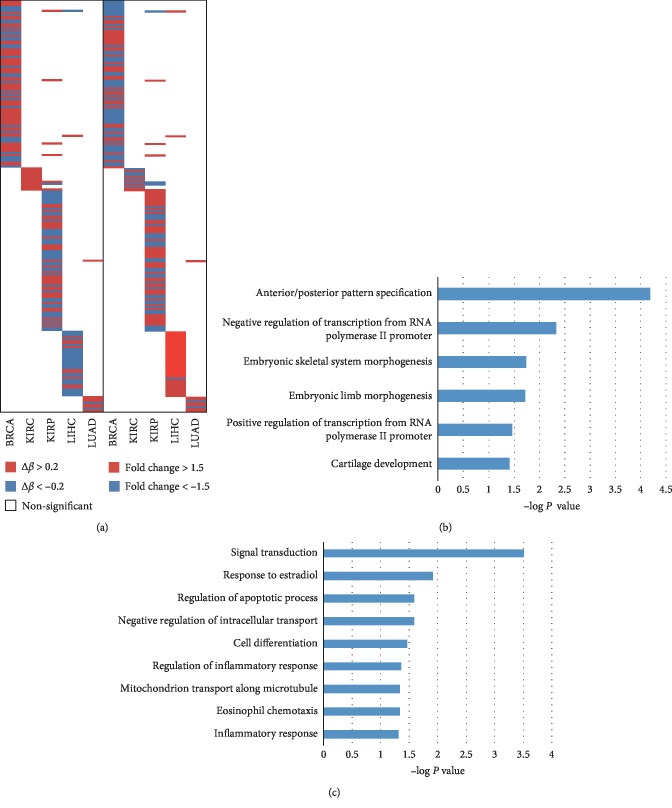
The significantly differentially methylated genes reveal distinct effects of *DNMT3B* expression. (a) The heat map of different expression genes contributed by *DNMT3B* across cancers. (b) Gene ontology analysis of hypermethylated genes (Δ*β* < –0.2). (c) Gene ontology analysis of hypomethylated genes (Δ*β* > 0.2).

Based on the analysis the DNA methylation disorders resulted from *DNMT3B*, different DNA methylation probes were obtained in high and low *DNMT3B* expression samples and then compared to the difference between tumor and normal. The number of altered methylation probes was varied from 26 to 234 across tumor types, and these changes may be contributed by high expression of *DNMT3B* (Supplementary [Supplementary-material supplementary-material-1]). These probes correspond to genes that were enriched in the different GO terms depending on the cancer types, such as regulation of cell differentiation in KIRC and signal transduction in KIRP (Supplementary [Supplementary-material supplementary-material-1]). Since the gene expression level was contributed by DNA methylation alteration in tumors, we overlapped the differential methylation and the differential expression genes data in cancer versus normal samples. 8 to 87 genes were screened out and were dysregulated with the differential methylation and mRNA across cancer types but few of them overlapped (Figure 6(a)). Functional annotation showed that the most frequently hypermethylated genes (Δ*β* > 0.2) across cancers were enriched in anterior/posterior pattern specification (Figure 6(b)), and the hypomethylated genes were in signal transduction (Figure 6(c)). Based on the calculated correlations between methylation and expression data, the highly anticorrelated genes were retained. After filtering and merging, thereinto, the hypermethylated-repressed and hypomethylated-activated genes are shown in Table 2 (Supplementary [Supplementary-material supplementary-material-1]).

### 3.6. Association between Metabolic Gene Expression and Oncogene or Tumor Suppressor Mutation

Metabolic reprogramming can be partly influenced by oncogenic driver events. To identify somatic alterations that potentially drive metabolic expression, we identified the top 3 oncogene/tumor suppressor with the highest frequency of mutations and assessed whether their mutation status correlated with the metabolic profile. THCA and KIRP were screened out because sample size with each 3 gene mutation was less than 20 (Supplementary [Supplementary-material supplementary-material-1]). For each cancer type, we performed the differential expression analysis of metabolic genes based on the two group samples with oncogene/tumor suppressor mutated or not (Supplementary [Supplementary-material supplementary-material-1]). *DNMT3B* was upregulated in BRCA, LIHC, and STAD in *TP53*-mutated conditions when compared against nonmutated samples. Upregulated expression *SLC2A1* was observed in LIHC and LUAD in *TP53*-mutated conditions. The other key metabolic genes related to DNA methylation and histone acetylation described in Table 1 were not associated with oncogenes or tumor suppressors within the scope of our study (Supplementary [Supplementary-material supplementary-material-1]).

We also analyzed the association between metabolic pathways and mutation patterns. Compared to other cancer types, most of metabolism pathways in BRCA were disordered with *TP53* and *MYC* mutation, respectively. In BRCA, LIHC, LUAD, HNSC, and STAD, groups with *TP53* mutations were associated with most of the genes related to nucleotide, lipid, and amino acid metabolisms. The other metabolic process associated with mutation patterns across cancer types is shown in Supplementary [Supplementary-material supplementary-material-1] and Supplementary [Supplementary-material supplementary-material-1].

## 4. Discussion

Dysregulation of metabolism and dysfunction of epigenetic modification is now the established feature of cancer. However, the mechanism of how metabolic gene rewriting influenced the global epigenetic modification levels in pan-cancer is not yet fully clear. We performed a pan-cancer analysis of transcriptome profiles covering 11 tumor types in a comprehensive collection of 2071 metabolic genes. We also elaborated on the altered metabolic genes which are involved in the dysfunction of epigenetic modifications including acetylation and methylation across cancers. *ACLY*, *SLC2A1*, *KAT2A*, and *DNMT3B* are the critical genes contributing to epigenetic dysfunction across cancers. Thereinto, *DNMT3B* is upregulated in 9 cancer types, and its higher expression showed a correlation with overall survival in 5 cancer types and it might contribute to the specific DNA methylation disorder. This result suggests a close link between *DNMT3B* expression, DNA methylation, and carcinogenesis.

Various metabolism pathways responsible for epigenetic modifications have been identified. One relevant pathway of metabolic gene rewiring and epigenetic landscape is the histone acetylation, which plays a critical role in regulating chromatin structure, thus participating in specific gene regulation [22]. A number of researches show that unstable histone acetylation is contributed by changed acetyl-group donor acetyl-CoA [23], which is a key metabolite in the mitochondria and cytoplasm, associated with breakdown of carbohydrates and fats via altered glycolysis and *β*-oxidation, respectively [24]. The acetyl-CoA synthesis is by ACSS2 [25] and ACLY [5] according to the different substrates. There is a similar or opposite trend for key gene expression, *ACSS2* is decreased in 5 cancers, and *ALCY* is found in 5 tumors compared to their normal samples. We consider that the citrate to the acyl-CoA pathway plays an important role in histone acetylation in the carcinogenesis of categories we studied. *ACLY* plays a critical role in determining the histone acetylation, and *ACLY* knockdown leads to apoptosis and growth suppression in breast cancer cells [26]. Inhibition of ACLY inhibits glucose-dependent histone acetylation to suppress glioblastoma cell proliferation [27]. *ACLY* was upregulated in COAD, COADREAD, HNSC, KIRC, and LIHC in the present study which indicates *ACLY* might promote tumor proliferation in these cancer types. Interestingly, the reduction in histone acetylation and defect of *SLC2A1* expression are observed in the *ACLY* knockdown cells [5]. Therefore, upregulated *ACLY* and *SLC2A1* in COAD, COADREAD, HNSC, KIRC, and LIHC might result in histone acetylation leading to selective regulation of genes involved in glucose metabolism. Thus, we speculate *ACLY* and *SLC2A1* may serve as critical linker of metabolic and histone acetylation in these cancer types. For metabolite analysis, lysine acetyltransferase 2A (KAT2A) is an affirmed histone acetyltransferase (HAT) that binds to acetyl-CoA and transfers the acetyl group to histones [28]. As expected, the results of our data show that the KAT2A gene has high expression in 9 cancer types, and it may be explained that *KAT2A* is important for controlling the gene expression program for adjusting histone acetylation in these cancer types. *KAT2A* over expression increases the extent of histone acetylation by interacting with *E2F1*, cyclin D1, and E1 promoters to promote the proliferation of lung cancer cell lines [29]. *KAT2A* knockdown leads to a significant reduction of DNA synthesis in cervical cancer cells by decreasing histone H3 acetylation in the *E2F1* promoter [30]. Our data show that the *KAT2A* has higher expression in 9 cancer types which indicates the increased histone acetylation level in specific gene promoters among these cancers to be involved in the development of cancer. Further study is needed to investigate *ACLY*, *SLC2A1*, and *KAT2A* in the histone acetylation function across cancer types.

Another epigenetic modification is the DNA methylation, changes in transmethylation influenced by altered methionine, choline, and folate. Unstable methylation usually relates to altered methyl donor [31]. In transmethylation reactions, methyl donor is finally provided by SAM which is produced from methionine by MAT [32]. Increased adenosylhomocysteinase (AHCY) gene expression will lead to an increase in SAM and aberrant DNA methylation [33]. *AHCY* knockdown leads to SAM depletion and proliferation rate reduction in hepatocellular carcinoma cells [34]. SAM is the methyl donor, and suppression of SAM leads to decreased DNA methylation and slows the growth of pancreatic cancer cells [35]. Upregulated *AHCY* gene expression is seen in BRCA, COAD, COADREAD, LIHC, LUAD, LUSC, and STAD that may indicate the transmethylation reactions have a more active state in these cancer types. The altered expression of *DNMTs,* encoding DNMTs (DNMT1, DNMT3a, and DNMT3B) could result in global changes of methylation in leukemia cells and breast cancer stem-like cells [36, 37], while *DNMT1*, *DNMT3A*, and *DNMT3B* are all upregulated in most cancer types (8, 6, and 9, respectively) in this study. Overexpressed *DNMT3A* contributes to the various methylation patterns and is a consequence of AML progression [37]. *DNMT1* promotes hypermethylation and downregulation of tumor suppressor gene *ISL1* which increases the tumor stem cell population in breast cancer cells [38]. However, only *DNMT3B* higher expression is correlated with poorer overall survival in BRCA, KIRP, KIRC, LIHC, and LUAD in the present study.

We then analyzed the DNA methylation level in 5 cancer types distinguished by high and low expression of *DNMT3B*. The relation between *DNMT3B* expression and methylation sites varies in cancer. Studies have shown that *DNMT3B* can induce DNA methylation in specific CpG islands in colorectal cancer [39] and it could also induce the distinct methylation level in different regions such as CpG and non-CpG [40]. Both hypermethylation and hypomethylation in gene promoters between high and low *DNMT3B* expression groups are found across cancers, which is consistent with the previous research [40]. We then choose the overlap genes which harbor differential methylation and expression; meanwhile, many of these genes are involved in different functional annotation that depends on the tissue types, suggesting that DNA methylation at different loci happens through DNMT3B in different cancers. Among these genes, we found numerous genes whose function in tumorigenesis and their expression associated with promoter hypermethylation or hypomethylation were well-documented in the literature. *ZNF154* and *AQP1*, whose hypermethylated patterns are biomarkers for distinguishing tumor from normal samples [41, 42], are downregulated and hypermethylated in BRCA and LUAD in our study. In addition, hypermethylation and downregulation of *PPP2R2B,* whose mRNA expression is in a DNMT-dependent manner [43], were found in BRCA. Loss of *DNMT3B* in the mouse model delayed the melanoma formation suggesting that other DNMTs do not adequately compensate for *DNMT3B* loss and suggesting nonredundant roles for DNMTs in melanoma and *DNMT3B*, in particular, may play specific, nonredundant roles [44, 45]. Inhibitions of DNMT3B as a novel therapeutic strategy for inhibiting the proliferation of carcinoma cells are being studied [43]. Together with these findings, it is suggested that *DNMT3B* with encoding protein DNMT3B and its potential methylation substrate genes would be the effective therapy targets to human cancer.

To understand the factors affecting the expression of metabolic genes at the present study, we have analyzed the association between oncogene/tumor suppressor mutation and metabolic gene expression in each cancer type. Several metabolic pathways, such as nucleotide, lipid, and amino acid metabolisms, are associated with specific mutation patterns across cancer types indicating that metabolic reprogramming may partly result from diverse oncogene/tumor suppressor alterations in different tumor contexts, which is also shown in the previous study [16]. Except for *SLC2A1* and *DNMT3B*, other genes related to histone acetylation and DNA methylation mentioned above were not associated with oncogene/tumor suppressor mutation at the present study. These results suggest that there might be other factors affecting the expression of metabolic genes, like TME, rather than just oncogene/tumor suppressor mutation. The expression of ACLY and ACSS2 is influenced by glucose concentration [27] and hypoxic conditions [46] in local microenvironment. TME modulates cancer cell metabolism and epigenetic modification contributing to tumor heterogeneity and therapeutic response through limited nutrient supply, acidic, hypoxia, and other characteristics [47–49]. Therefore, it is necessary to combine metabolic phenotype, epigenetic modification, genomic landscape, and local TME to reveal the relationship between metabolic genes and epigenetics modification in pan-cancer, which may help overcome therapy resistance in cancer patients and guide the rational design of combinational therapies targeting both tumor cells and microenvironment components.

## 5. Conclusion

In summary, our research clarifies some key metabolic genes, *ACLY*, *SLC2A1*, *KAT2A*, and *DNMT3B*, which are involved in epigenetic regulation indirectly in tumors. The uniqueness of our study is that it is first report that presents metabolic gene reprogramming as a factor influencing epigenetic regulation across human pan-cancers. We found some potential genes' methylation and expression in different cancer types influenced by *DNMT3B* whose expression is strongly relevant to the patient overall survival. Besides, the connection was identified linking oncogenes and tumor suppressors with deregulated cancer cell metabolism. The metabolic phenotype affected by TME should also be gotten attention. Thus, targeting these tumor metabolic genes might reverse epigenetic dysregulation and improve the effect of targeted therapy. Moreover, the combinational therapies targeting metabolic phenotype and epigenetic modification with genomic landscape and local TME can more effectively eradicate tumor cells from the patients and improve the quality of life.

## Figures and Tables

**Table 1:** List of the critical different expression genes of categories we studied in the specific types of cancer.

Terms	Genes	Upregulation cancer types	Downregulation cancer types
Methylation	*SLC19A1*	BRCA/COAD/COADREAD/HNSC/LIHC/STAD	LUAD/LUSC/THCA
	*SLC44A4*	BRCA/KIRP/LIHC/LUAD	COAD/COADREAD/HNSC/KIRC/LUSC
	*DHFR*	COAD/COADREAD/HNSC/LUAD/LUSC/STAD	
	*CHDH*	COADREAD/STAD	HNSC/KIRC/LUSC
	*BHMT*		KIRC/KIRP/LIHC
	*MTHFD1*	COAD/COADREAD/HNSC/LUAD/LUSC/STAD	LIHC
	*MTHFD2*	BRCA/COAD/COADREAD/HNSC/KIRC/LIHC/LUAD/LUSC/STAD	
	*AHCY*	BRCA/COAD/COADREAD/LIHC/LUAD/LUSC/STAD	KIRC/KIRP
	*MAT1A*	BRCA/COAD/COADREAD/LUAD/LUSC	LIHC
	*MAT2A*	LIHC	THCA
	*DNMT1*	BRCA/COAD/COADREAD/HNSC/LIHC/LUAD/LUSC/STAD	
	*DNMT3A*	BRCA/HNSC/LIHC/LUAD/LUSC/STAD	
	*DNMT3B*	BRCA/COAD/COADREAD/HNSC/KIRP/LIHC/LUAD/LUSC/STAD	

Acetylation	*SLC2A1*	BRCA/COAD/COADREAD/HNSC/KIRC/LIHC/LUAD/LUSC/STAD	
	*HK2*	HNSC/KIRC/KIRP/LIHC/LUSC/STAD	COAD/COADREAD
	*PKM2*	BRCA/COAD/COADREAD/HNSC/LIHC/LUAD/LUSC/STAD	
	*PFKP*	BRCA/HNSC/KIRC/KIRP/LUAD/LUSC/STAD	
	*SLC25A20*		BRCA/COAD/COADREAD/HNSC/KIRC/KIRP/LUSC/STAD
	*FASN*	COAD/COADREAD/LIHC/STAD	
	*ACACA*	COAD/COADREAD/LIHC/STAD	KIRC
	*ACLY*	COAD/COADREAD/HNSC/KIRC/LIHC	
	*ACSS2*		BRCA/COAD/COADREAD/KIRC/LUSC
	*KAT2A*	COAD/COADREAD/HNSC/LIHC/LUAD/LUSC/KIRC/KIRP/STAD	

Note: the blank content means the genes had no upregulation expression or downregulation in one particular cancer type.

**Table 2:** The different expression genes with hypermethylation or hypomethylation in the specific types of cancer.

Cancer types	Probe	Δ*β*	Gene	Fold change	*P* value	*r*	*P* value
BRCA	cg05661282	0.328652	*ZNF154*	–2.51052	0	–0.5733	4.31*E* – 69
	cg02938205	0.313603	*CCDC36*	–4.76933	0	–0.25194	1.03*E* – 12
	cg11417025	0.340146	*SOSTDC1*	–3.43449	5.82*E* – 11	–0.45553	4.54*E* – 41
	cg17116120	0.203629	*COX4I2*	–2.06691	0	–0.26144	1.31*E* – 13
	cg06087421	0.206592	*TAL1*	–3.82039	0	–0.24326	6.26*E* – 12
	cg06363129	0.297534	*SOSTDC1*	–3.43449	5.82*E* – 11	–0.49109	2.09*E* – 48
	cg03757145	0.274106	*CDKL2*	–2.28953	2.5*E* – 08	–0.63537	4.45*E* – 89
	cg12042659	0.231631	*ZNF132*	–2.00658	0	–0.48716	1.49*E* – 47
	cg07220448	0.29423	*SOSTDC1*	–3.43449	5.82*E* – 11	–0.45009	5.08*E* – 40
	cg10168635	0.267563	*C2orf88*	–5.55411	0	–0.32566	1.18*E* – 20
	cg14625175	0.405266	*HOXA10*	–2.7302	0	–0.2537	7.06*E* – 13
	cg14263942	0.281991	*CDKL2*	–2.28953	2.5*E* – 08	–0.63136	1.19*E* – 87
	cg18868483	0.301089	*B3GAT2*	–1.54216	4.51*E* – 05	–0.22273	3.44*E* – 10
	cg01454592	0.245132	*CCDC36*	–4.76933	0	–0.24726	2.75*E* – 12
	cg10344081	0.294167	*CDKL2*	–2.28953	2.5*E* – 08	–0.63967	1.23*E* – 90
	cg06126713	0.395028	*SOSTDC1*	–3.43449	5.82*E* – 11	–0.44907	7.94*E* – 40
	cg24432073	0.20478	*CDKL2*	–2.28953	2.5*E* – 08	–0.62519	1.73*E* – 85
	cg02085507	0.372686	*TRIP10*	–1.54805	0	–0.20553	7.39*E* – 09
	cg05308656	0.253266	*ARL4C*	–1.64326	6.71*E* – 13	–0.52274	1.06*E* – 55
	cg11270393	0.308156	*ITPRIPL1*	–5.73674	0	–0.39404	2.9*E* – 30
	cg12506930	0.323833	*ZNF154*	–2.51052	0	–0.57088	2.14*E* – 68
	cg07519235	0.459351	*GPRC5B*	–2.5035	0	–0.49936	3.06*E* – 50
	cg21113446	0.215296	*PPP2R2B*	–3.10098	0	–0.33333	1.29*E* – 21
	cg02324432	0.253122	*KLHL2*	–1.79165	0	–0.23855	1.62*E* – 11
	cg04203238	0.274658	*PROM1*	–1.68571	0.000897	–0.64459	1.9*E* – 92
	cg04243822	0.216055	*C1QTNF1*	–3.30512	0	–0.22631	1.76*E* – 10
	cg00680551	0.424494	*NCALD*	–3.00007	0	–0.37035	1.14*E* – 26
	cg27049766	0.32279	*ZNF154*	–2.51052	0	–0.61756	6.93*E* – 83
	cg08816590	0.288288	*PDE1B*	–3.70157	0	–0.30695	2.05*E* – 18
	cg14988503	0.210715	*CDKL2*	–2.28953	2.5*E* – 08	–0.58357	4.19*E* – 72
	cg18236571	0.263708	*PABPC4L*	–1.69422	2.54*E* – 09	–0.48483	4.73*E* – 47
	cg18085435	–0.20795	*ATP8B1*	1.700614	8.59*E* – 08	–0.56131	1.05*E* – 65
	cg01062470	–0.2649	*KIF24*	2.804288	0	–0.30429	4.14*E* – 18
	cg04567302	–0.25404	*SLC44A4*	5.457885	0	–0.6607	1.26*E* – 98
	cg16702815	–0.32459	*AGR3*	5.086635	3.09*E* – 12	–0.69724	3.7*E* – 114
	cg10173620	–0.29247	*ABHD2*	1.920326	0.000667	–0.36501	6.75*E* – 26
	cg11819637	–0.26298	*THPO*	3.885221	1.36*E* – 06	–0.67328	1*E* – 103
	cg08960448	–0.33095	*SEPT12*	5.27739	7.67*E* – 05	–0.30054	1.1*E* – 17
	cg27530053	–0.20353	*FAM83E*	3.858755	8.11*E* – 09	–0.458	1.5*E* – 41
	cg23631538	–0.3347	*DENND2D*	1.578295	8.5*E* – 13	–0.43718	1.31*E* – 37
	cg12633764	–0.29531	*MAPT*	1.804312	4.72*E* – 05	–0.72534	8.3*E* – 128

KIRC	cg27026192	0.214365	*KIFC3*	–1.57694	0	–0.36061	2.72*E* – 11
	cg02993070	0.296156	*FERMT2*	–1.58269	3.77*E* – 15	–0.26413	1.59*E* – 06
	cg02852670	0.260053	*KLHL33*	–3.21637	0	–0.25921	2.52*E* – 06

KIRP	cg11800117	0.293577	*C11orf75*	–1.50718	3.06*E* – 05	–0.26745	7.16*E* – 06
	cg20962532	0.209952	*KCNJ1*	–32.1899	0	–0.26319	1.01*E* – 05
	cg11968091	0.261459	*ODZ4*	–2.24539	0.000389	–0.30657	2.26*E* – 07
	cg05227215	0.41792	*CXXC5*	–2.04975	0	–0.25958	1.35*E* – 05
	cg04858586	0.269997	*RABGAP1L*	–2.15075	5.35*E* – 13	–0.21864	0.000266
	cg03648711	0.255349	*ODZ4*	–2.24539	0.000389	–0.2788	2.77*E* – 06
	cg24277788	0.243307	*ACSL1*	–2.44389	1.27*E* – 14	–0.33942	8.17*E* – 09
	cg03883256	0.265289	*USP2*	–3.63204	0	–0.21891	0.000261
	cg10416846	0.299733	*PNOC*	–4.19665	2.27*E* – 08	–0.25688	1.67*E* – 05
	cg24127861	0.207238	*REC8*	–2.2299	0.000653	–0.31031	1.58*E* – 07
	cg15974053	0.242659	*HSD17B14*	–2.21561	2.94*E* – 05	–0.73157	3.69*E* – 47
	cg16530498	0.222937	*HSD17B14*	–2.21561	2.94*E* – 05	–0.74962	1.14*E* – 50
	cg19414598	0.214085	*DMC1*	–1.57414	0.010418	–0.46463	4.44*E* – 16
	cg15731317	–0.37473	*SYTL2*	1.895209	0.000186	–0.50345	5.16*E* – 19
	cg00583003	–0.28896	*SPP1*	2.483728	3.64*E* – 06	–0.2564	1.73*E* – 05
	cg21622977	–0.22364	*RBPMS*	1.793989	3.22*E* – 08	–0.49531	2.29*E* – 18
	cg17660833	–0.53805	*HRH1*	4.616294	2.92*E* – 08	–0.33817	9.35*E* – 09
	cg20306842	–0.21944	*CREB5*	7.099205	1.84*E* – 10	–0.40814	2.01*E* – 12
	cg23031196	–0.31756	*SLC38A1*	1.620018	0.000342	–0.37927	8.38*E* – 11
	cg01944226	–0.28436	*SLC16A3*	4.39414	8.6*E* – 06	–0.76961	6.36*E* – 55
	cg09182455	–0.41989	*CORO1C*	2.191563	4*E* – 11	–0.22222	0.000209
	cg01812894	–0.26035	*ALDH1A1*	2.206084	0.000791	–0.2834	1.86*E* – 06
	cg01942558	–0.4858	*TNFAIP6*	45.14506	1.33*E* – 05	–0.68008	1.51*E* – 38
	cg09122223	–0.34216	*IL18*	2.298725	1.94*E* – 07	–0.24966	2.91*E* – 05
	cg16929739	–0.38802	*HRH1*	4.616294	2.92*E* – 08	–0.32623	3.25*E* – 08
	cg09727050	–0.39502	*TNFAIP6*	45.14506	1.33*E* – 05	–0.78361	3.61*E* – 58
	cg07042532	–0.32291	*CREB5*	7.099205	1.84*E* – 10	–0.62039	1.54*E* – 30
	cg13445177	–0.29628	*S100A10*	1.832289	4.18*E* – 08	–0.34141	6.61*E* – 09
	cg14333454	–0.30823	*SFN*	11.18657	0.034773	–0.7839	3.08*E* – 58
	cg08778148	–0.2173	*TFPI*	2.08801	0.00022	–0.44876	5.55*E* – 15
	cg14578677	–0.23981	*TLR6*	2.307439	0.007805	–0.44887	5.46*E* – 15
	cg12669354	–0.35182	*CD151*	1.854554	1.27*E* – 11	–0.37366	1.66*E* – 10
	cg00449608	–0.3179	*GPR45*	3.093416	0.00448	–0.44012	2.08*E* – 14
	cg26864526	–0.36952	*HRH1*	4.616294	2.92*E* – 08	–0.53238	1.86*E* – 21
	cg10183885	–0.26987	*SLC16A3*	4.39414	8.6*E* – 06	–0.8219	1.96*E* – 68
	cg06836480	–0.24006	*DHRS9*	9.536973	0.012336	–0.23825	6.8*E* – 05
	cg11702866	–0.44443	*HRH1*	4.616294	2.92*E* – 08	–0.29902	4.58*E* – 07
	cg06457736	–0.44623	*HRH1*	4.616294	2.92*E* – 08	–0.31287	1.23*E* – 07
	cg24258705	–0.47246	*MET*	4.732257	1.75*E* – 08	–0.63402	3.26*E* – 32
	cg15717250	–0.34571	*UPK1B*	4.304701	0.004226	–0.66398	3.33*E* – 36
	cg03489712	–0.29538	*ZYX*	1.99858	3.19*E* – 10	–0.22033	0.000237
	cg09950681	–0.33674	*PKP3*	2.143401	0.047341	–0.60723	5.34*E* – 29
	cg14550985	–0.33893	*RIN1*	5.119397	2.06*E* – 10	–0.68575	2.09*E* – 39
	cg21950166	–0.36612	*SFN*	11.18657	0.034773	–0.71606	2.3*E* – 44
	cg06720467	–0.34964	*SFN*	11.18657	0.034773	–0.55134	3.43*E* – 23
	cg20992002	–0.30043	*PKP3*	2.143401	0.047341	–0.6543	7.26*E* – 35
	cg02872476	–0.23267	*DBNDD1*	3.125374	4.05*E* – 08	–0.66917	6.07*E* – 37

LIHC	cg21831174	0.218134	*MASP1*	–2.16898	0	–0.35207	1.19*E* – 11
	cg04951797	–0.28077	*SGOL1*	19.52393	5.5*E* – 07	–0.62482	2.7*E* – 39
	cg17336139	–0.25975	*PRAME*	231.9457	0.018915	–0.36725	1.29*E* – 12
	cg23213217	–0.24898	*DEGS1*	2.328115	2.56*E* – 08	–0.33786	8.59*E* – 11
	cg11225751	–0.21878	*PRAME*	231.9457	0.018915	–0.46426	4.12*E* – 20
	cg06442489	–0.34313	*ZSCAN18*	1.965757	0.001058	–0.28886	3.75*E* – 08
	cg20441902	–0.22001	*FUT2*	14.81239	0.012527	–0.54902	6.06*E* – 29
	cg25060890	–0.22045	*RPS6KC1*	2.58206	2.81*E* – 11	–0.39031	3.5*E* – 14
	cg03368046	–0.20541	*FAM186A*	4.276926	0.000748	–0.24311	4.2*E* – 06
	cg23924737	–0.25896	*MRPS23*	2.825878	4*E* – 15	–0.40317	4.11*E* – 15
	cg01999046	–0.30348	*TRAF2*	3.59974	4*E* – 15	–0.48642	3.45*E* – 22
	cg03212674	–0.22852	*CLK2*	3.219276	7.32*E* – 13	–0.26115	7.25*E* – 07
	cg02516101	–0.21135	*CSNK1E*	2.573716	5*E* – 09	–0.43473	1.44*E* – 17
	cg07303143	–0.37162	*KIAA1143*	1.713539	2.33*E* – 07	–0.4433	2.79*E* – 18

LUAD	cg04372674	0.214314	*AQP1*	–2.70913	7.09*E* – 14	–0.6127	3.66*E* – 49
	cg25075794	0.226309	*AQP1*	–2.70913	7.09*E* – 14	–0.60885	2.09*E* – 48
	cg02571816	0.216135	*PPP1R14A*	–3.40955	0	–0.34411	2.41*E* – 14

## Data Availability

The data used to support the findings of this study are available from the Cancer Genome Atlas and the Gene Expression Omnibus database freely.

## Abbreviations

TCGA: The cancer genome atlas
GEO: The gene expression omnibus
ACLY: ATP citrate lyase
SLC2A1: Solute carrier family 2 member 1
KAT2A: Lysine acetyltransferase 2A
DNMT1: DNA methyltransferase
BRCA1: DNA repair associated
TP53: Tumor protein P53
CDH13: Cadherin 13
BRCA: Breast invasive carcinoma
COAD: Colon adenocarcinoma
COADREAD: Colon rectum adenocarcinoma
HNSC: Head and neck squamous cell carcinoma
KIRC: Kidney renal clear cell carcinoma
KIRP: Kidney renal papillary cell carcinoma
LIHC: Liver hepatocellular carcinoma
LUAD: Lung adenocarcinoma
LUSC: Lung squamous cell carcinoma
STAD: Stomach adenocarcinoma
THCA: Thyroid carcinoma
EGs: Expressed genes
DEGs: Differentially expressed genes
MDEGs: Metabolic differential expression genes
FDR: False discovery rate
SAM: S-adenosylmethionine
DNMT1: DNA methyltransferase 1
DNMT3A: DNA methyltransferase 3 alpha
DNMT3B: DNA methyltransferase 3 beta
AHCY: Adenosylhomocysteinase
Acetyl-CoA: Acetyl coenzyme A
ACLY: ATP citrate lyase
KAT2A: Lysine acetyltransferase 2A
SLC2A1: Solute carrier family 2 member 1
ZNF154: Zinc finger protein 154
AQP1: Aquaporin
PPP2R2B: Protein phosphatase 2 regulatory subunit beta
E2F1: E2F transcription factor 1
ISL1: ISL LIM homeobox 1.

## Supplementary Materials

Supplementary Materials Supplementary Table 1: the number of sample information and differential expression genes for 11 different cancer types and 5 GEO data projects. Supplementary Table 2: the differential expression gene list and its fold change in 11 cancer types compared with normal samples. Supplementary Table 3: the metabolic genes and the differential expression of metabolic genes. Supplementary Table 4: the function annotations of MDEGs. Supplementary Table 5: the critical different expression genes related to epigenetic modification in the specific types of cancer. Supplementary Table 6: the genes with different expression and methylation influenced by *DNMT3B* expression. Supplementary Table 7: gene ontology analysis of differential expression and methylation genes. Supplementary Table 8: the correlation between expression and methylation of specific genes. Supplementary Table 9: the oncogene/tumor suppressor mutation frequency and the sample size with mutation across cancer types. Supplementary Table 10: the differential expression MDEGs in mutated samples compared with non-mutated samples across cancer types. Supplementary Table 11: the pathway enrichment analysis in each mutation group. Supplementary Figure 1: relationships among metabolic pathways, cancer types, and oncogene/suppressor gene mutations.
