# Supplementary material for: Integrative Analysis Reveals Comprehensive Altered Metabolic Genes Linking with Tumor Epigenetics Modification in Pan-Cancer
Source: Biomed Res Int. 2019 Nov 7;2019:6706354. doi: 10.1155/2019/6706354 (PMC6881592; doi:10.1155/2019/6706354)
Supplement: Supplementary Materials — Supplementary Table 1: the number of sample information and differential expression genes for 11 different cancer types and 5 GEO data projects. Supplementary Table 2: the differential expression gene list and its fold change in 11 cancer types compared with normal samples. Supplementary Table 3: the metabolic genes and the differential expression of metabolic genes. Supplementary Table 4: the function annotations of MDEGs. Supplementary Table 5: the critical different expression genes related to epigenetic modification in the specific types of cancer. Supplementary Table 6: the genes with different expression and methylation influenced by DNMT3B expression. Supplementary Table 7: gene ontology analysis of differential expression and methylation genes. Supplementary Table 8: the correlation between expression and methylation of specific genes. Supplementary Table 9: the oncogene/tumor suppressor mutation frequency and the sample size with mutation across cancer types. Supplementary Table 10: the differential expression MDEGs in mutated samples compared with non-mutated samples across cancer types. Supplementary Table 11: the pathway enrichment analysis in each mutation group. Supplementary Figure 1: relationships among metabolic pathways, cancer types, and oncogene/suppressor gene mutations. [file 6706354.f1.zip › 6706354.f1/Supplementary Figure 1.docx]

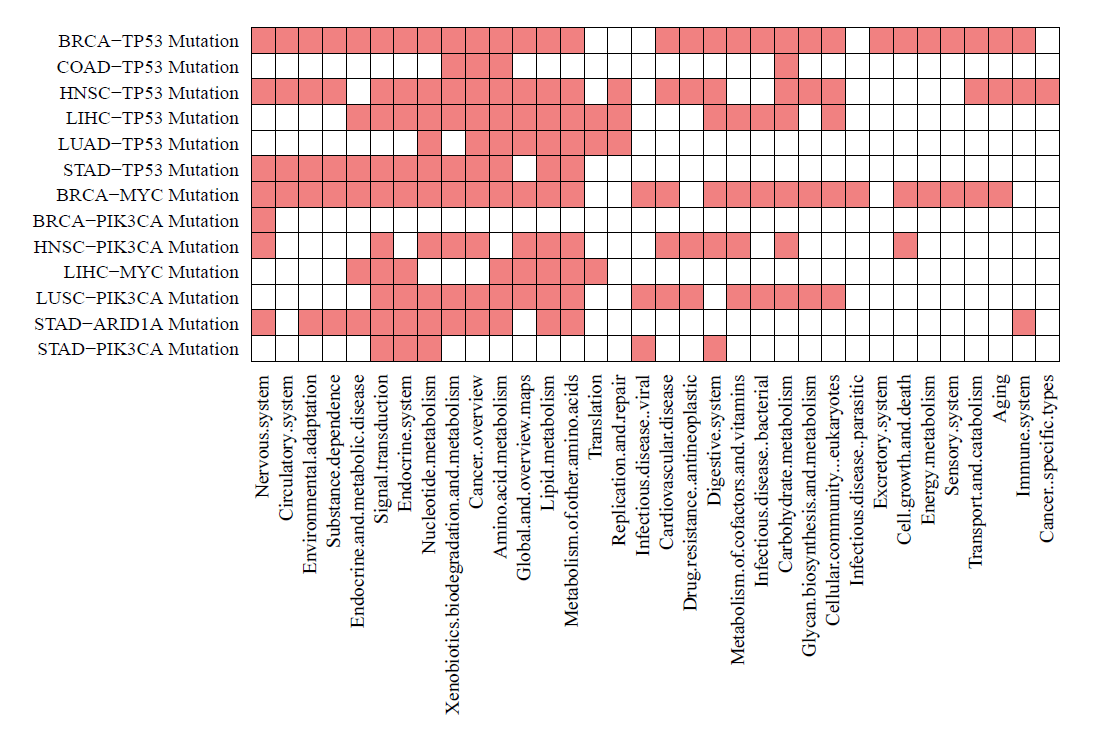


Supplementary Figure1. Relationships among metabolic pathways, cancer types, and oncogene/suppressor gene mutations.

Heatmap shows the samples with deregulated processes associated with specific mutations. The most frequently mutated driver genes are shown with each cancer type.

Red represents the genes in metabolic pathways is significantly differentially expressed in samples with specific oncogene/suppressor gene mutations. White represents non-significant difference.
